# The efficacy and safety of traditional Chinese medicine treating diabetic cardiomyopathy: A protocol for systematic review and meta-analysis

**DOI:** 10.1097/MD.0000000000031269

**Published:** 2022-11-25

**Authors:** Shuo Han, Yuan Hou, Huaman Liu, Quanlin Zhao

**Affiliations:** a Zibo Branch of the 960th Hospital of the Chinese People’s Liberation Army, Zibo, China; b Shandong University of Chinese Medicine, Jinan, China; c The Affiliated Hospital of Shandong University of Traditional Chinese Medicine, Jinan, China.

**Keywords:** diabetic cardiomyopathy, meta-analysis, traditional Chinese medicine

## Abstract

Diabetic cardiomyopathy, secondary to diabetes, is the main cause of death in patients with diabetes. In China, traditional Chinese medicine has achieved good performance in treating diabetic cardiomyopathy. However, to date, no systematic review or meta-analysis has been published on the treatment of diabetic cardiomyopathy by traditional Chinese medicine.

**Methods:**

This study strictly followed the preferred guidelines for systematic review. Two researchers searched seven databases: EMbase, PubMed, Web of Science, Cochrane Library, China National Knowledge Infrastructure, Chinese Scientific Journal Database, and WANFANG Database. The retrieval time limit ranged from the establishment of the database to August 2022. All clinical randomized controlled trials that met the inclusion and exclusion criteria were included in this study. Statistical analysis was performed using RevMan 5.3.

**Results:**

This study analyzed the clinical efficacy and safety of traditional Chinese medicine in the treatment of diabetic cardiomyopathy.

**Conclusion:**

The results of this study provide evidence-based medical evidence for the clinical use of traditional Chinese medicine in the treatment of diabetic heart disease in the future.

## 1. Introduction

Diabetic cardiomyopathy (DC) is a heart disease with abnormal myocardial structure and function caused by diabetes in the absence of cardiac risk factors such as hypertension, coronary artery disease, and heart valve disease.^[1,2]^ Glycolipid toxicity,^[3]^ myocardial cell calcium imbalance,^[1]^ mitochondrial damage,^[4]^ chronic inflammation,^[5]^ oxidative stress,^[6]^ pyroptosis^[7]^ and myocardial fiber^[8]^ are possible pathological mechanisms at present. The onset of DC is insidious, and only manifests as molecular-level damage to myocardial cells at the early stage, without obvious clinical manifestations. In the middle and late stages of the disease, DC can manifest as continuous aggravation of myocardial cell damage, extensive necrosis of small myocardial cells, and myocardial fibrosis, eventually leading to irreversible myocardial structural remodeling and cardiac function decline. The main clinical manifestations include angina pectoris, arrhythmia, cardiogenic shock, and heart failure.^[9,10]^

Epidemiological studies showed that by 2021, diabetic patients in the world reached 537 million,^[11]^ accounting for 10.5% of the global population. It is proved that 19% to 26% of DM patients will develop heart failure,^[1]^ and 50% to 80% of DM patients will die from cardiovascular complications.^[12]^ Current treatment measures for DC are relatively limited, and no antidiabetic drugs except sodium glucose cotransporter 2 inhibitors have obvious cardiovascular benefits. Intensive hypoglycemia cannot effectively prevent the occurrence of cardiovascular events in DM patients^[13]^ but increases the risk of heart failure. In addition, effective treatment measures for DC cardiotoxicity have not been established,^[14]^ and there are no specific drug recommendations in the guidelines. The search for effective therapeutic targets for DC remains an urgent issue. In recent years, traditional Chinese medicine compounds and proprietary Chinese medicines have shown advantages in the treatment of DC because of their multichannel, multitarget, and slight side effects. A large amount evidence suggests that traditional Chinese medicine may be a potential drug for the treatment of DC.^[15]^

Randomized controlled trials published in journals were selected for this study. The aim of this study was to conduct a high-quality systematic evaluation of the clinical efficacy and safety of traditional Chinese medicine combined with Western medicine in the treatment of DC in order to provide more credible evidence-based medical evidence for the clinical application of traditional Chinese medicine in the treatment of DC.

## 2. Methods

### 2.1. Study methods and registration

The protocol was registered with PROSPERO (registration number: CRD42022352653; https://www.crd.york.ac.uk/prospero/display_record.php?ID=CRD42022352653). The protocol was carried out strictly in accordance with the preferred guidelines for systematic reviews. All data in this study were derived from published studies and therefore do not require ethical approval.

### 2.2. Study inclusion criteria

#### 2.2.1. Types of studies.

Randomized controlled studies were published in Chinese and English, with no restrictions on time, region, and follow-up time.

#### 2.2.2. Types of patients.

Patients with a definitive diagnosis of DC^[16,17]^ without restrictions on medical records, age, sex, race, or nationality.

#### 2.2.3. Types of interventions.

The control group received Western medicine treatment (including hypoglycemic, lipid-lowering, blood pressure control, and cardiac function improvement), while the experimental group was given traditional Chinese medicine (including traditional Chinese medicine decoction, Chinese patent medicine, and traditional Chinese medicine injection) on the basis of the control group.

#### 2.2.4. Types of outcome measures.

##### 2.3.4.1. Primary outcomes.

The primary outcome is clinical effectiveness rate.

##### 2.3.4.2. Secondary outcomes.

Other outcomes included left ventricular ejection fraction, the ratio of peak early diastolic blood flow velocity to peak late diastolic blood flow velocity (E/A), fasting blood glucose, 2h postprandial blood glucose, glycosylated hemoglobin, total cholesterol, triglyceride, low-density lipoprotein cholesterol, and incidence of adverse events.

### 2.3. Study exclusion criteria

Duplicate publications. Incomplete or incorrect experimental data. The outcome observation indicators formulated in this study were extracted. Intervention measures did not meet these requirements.

### 2.4. Search methods

#### 2.4.1. Search database.

Seven databases, including EMbase, PubMed, Web of Science, Cochrane Library, China National Knowledge Infrastructure, Chinese Scientific Journal Database, and WANFANG Database, were searched using a computer. The retrieval time limit was set from the establishment of the database to October 2022.

#### 2.4.2. Search terms.

Search terms include: “traditional Chinese medicine,” “Chinese herbal medicine,” “prescription,” “soup,” “powder,” “drink,” “injection,” “capsule,” “pill,” “granule,” “diabetic cardiomyopathy,” and “DC”.

#### 2.4.3. Search strategy.

A detailed example of the PubMed search strategy is shown in Table 1.

**Table 1:** Search strategy for the PubMed database.

Number	Search items
#1	Traditional Chinese medicine
#2	Chinese herbal medicine
#3	Prescription
#4	Soup
#5	Powder
#6	Drink
#7	Injection
#8	Capsule
#9	Pill
#10	Granule
#11	#1 or #2 or #3 or #4 or #5 or #6 or#7 or #8 or #9 or #10
#12	Diabetic cardiomyopathy
#13	DC
#14	#12 or #13
#15	Randomized controlled trial
#16	RCT
#17	#15 or #16
#18	#11 or #14 or #17

DC = Diabetic cardiomyopathy.

### 2.5. Study selection and data extraction

#### 2.5.1. Study selection.

**Figure 1.**
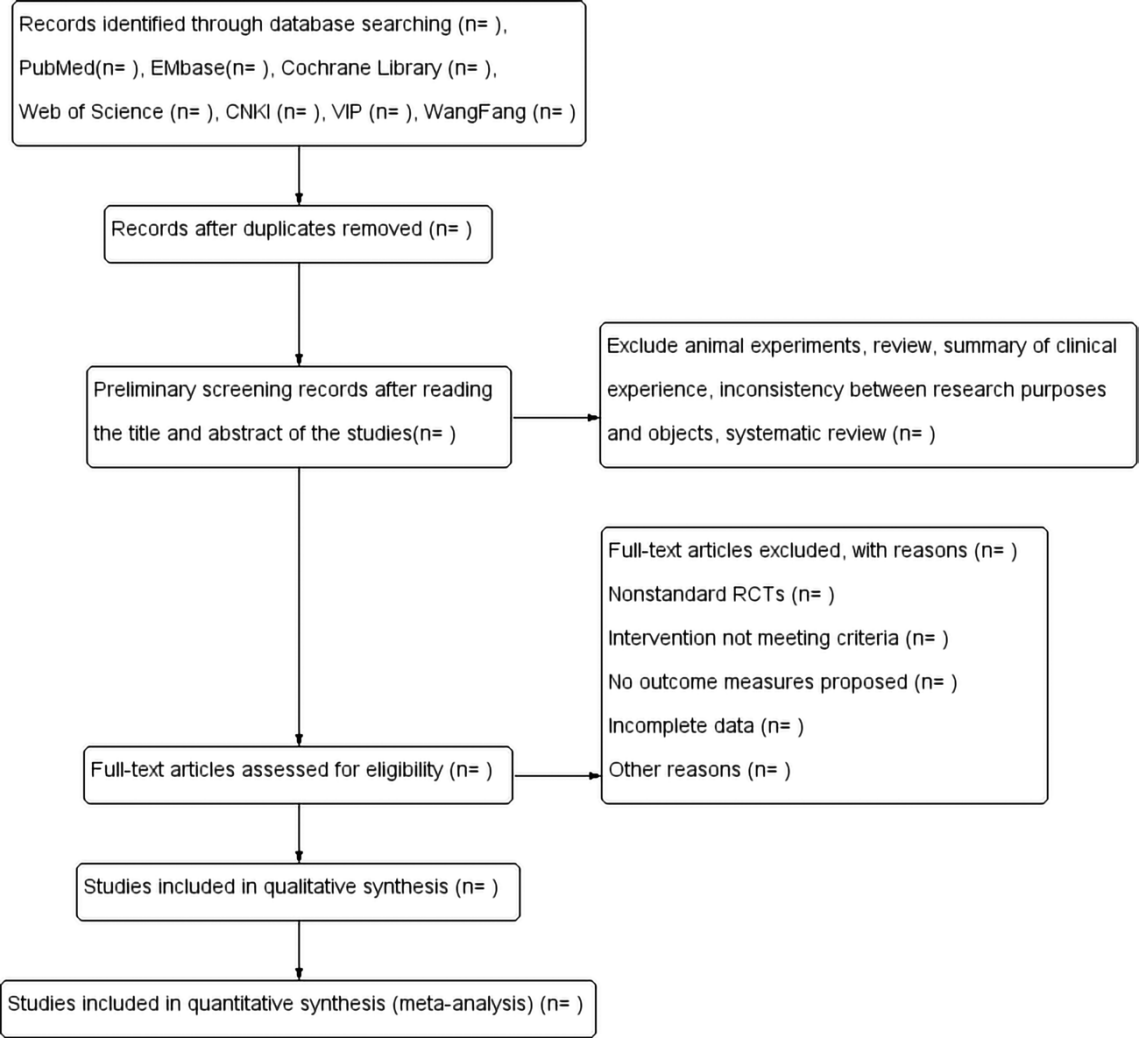
Studies Retrieval Flowchart.

Two independent researchers (HS and HY) searched and filtered the literature. By reading the title, abstract, and full text, combined with the inclusion and exclusion criteria, the literature that could be included in this meta-analysis will be determined, and the inclusion results will be cross-checked. If there is any disagreement, a third researcher (LHM) will assist with the judgment. The selection process for the studies is shown in the flowchart (Fig. 1).

#### 2.5.2. Data extraction.

A data extraction table was designed using Microsoft Excel after filtering the data. The data were extracted by two researchers (HS and HY), and then the data characteristics of the final included literature were sorted out, mainly including: study characteristics (including literature author, country, publication year, original inclusion and exclusion criteria, and sample size), characteristics of the research subjects (including sex, average age, medical history), clinical trial methods (including intervention measures, course of treatment), clinical trial methods (including intervention measures, courses of treatment), and outcome observation indicators (including total clinical response rate, cardiac function, blood sugar, blood lipids, adverse events, and follow-up results). If the information is incomplete, the original author of the article will be contacted by email or phone. Disagreements were resolved through consultation with a third researcher.

### 2.6. Missing data processing

If data were missing from the included studies, we contacted the corresponding authors for missing data and additional information. If the corresponding author does not reply after one month, we will consider deleting the data, or analyzing the available information and perform a sensitivity analysis to reduce the potential impact of the data on the results of this study.

### 2.7. Risk of bias assessment

Cochrane collaboration’s tool will be utilized for assessing risk of bias provided by the Cochrane Handbook for Systematic Reviews of Interventions to assess the risk of bias in the included literature; the literature will be rated as High risk of bias, Unclear risk of bias, Low risk of bias. The assessment included six domain-based evaluations, and the details are listed in Table 2. Two researchers (HS and HY) evaluated each article separately, and a third researcher (LHM) was consulted in cases of disagreement. Bias results are presented with the risk of bias ratio plot and the risk of bias assessment table.

**Table 2:** The Cochrane collaboration’s tool for assessing risk of bias.

Domain-based evaluation	Comment content
Selection bias	Random sequence generation
	Allocation concealment
Performance bias	Blinding of participants and personnel
Detection bias	Blinding of outcome assessment
Attrition bias	Incomplete outcome data
Reporting bias	Selective reporting
Other bias	

### 2.8. Data synthesis and analysis

Statistical processing was performed using Review Manager software (version 5.3), and the results are displayed as a forest plot.

#### 2.8.1. Statistical indicators.

The count data were expressed as odds ratios, and the measurement data were expressed as weighted mean differences. All effect sizes were expressed with 95% confidence intervals, and *P* < .05 was considered statistically significant.

#### 2.8.2. Assessment of heterogeneity.

*I*^2^ test was used for statistical heterogeneity tests. If *P* ≥ .1, *I*^2^ ≤ 50%, it indicated that the heterogeneity of each study was small, and a fixed effect model was used for analysis; if *P* < .1, *I*^2^ > 50%, indicating that the heterogeneity was large, and a random effect model was selected.

### 2.9. Supplementary analysis

#### 2.9.1. Subgroup analysis.

When there is large heterogeneity, subgroup analysis (e.g., age, intervention, and duration of treatment) can be used to analyze the source of heterogeneity.

#### 2.9.2. Sensitivity analysis.

Sensitivity analyses will be performed if low-quality studies are included. In addition, sensitivity analysis was performed when there was large heterogeneity between the studies.

### 2.10. Assessment of reporting biases

A funnel plot was used to assess the presence of reporting bias.

## 3. Discussion

DC is a major adverse factor that affects the prognosis and survival of patients with diabetes. Owing to the complex pathogenesis and non-obvious early stage symptoms, the diagnosis and treatment of DC have brought great difficulties. In recent years, with the extensive development of basic research on Chinese herbal medicine, the mechanism of action of Chinese medicine in DC treatment has been scientifically confirmed.^[15]^ Breviscapine can interfere with the Nrf2 signaling pathway to improve the cardiac function of the DC mouse model^[18]^; Andrographis paniculata Ester protects the DC myocardium by down-regulating the expression of signaling pathways such as IL-6 and NF-κB^[19]^; bakuchiol can inhibit cardiomyocyte fibrosis.^[20]^ A large number of clinical trials of traditional Chinese medicine in the treatment of DC have been carried out, and the effectiveness of traditional Chinese medicine in the treatment of DC has been confirmed. However, no high-quality systematic reviews and meta-analyses related to DC have been published, and their efficacy and safety have not been widely recognized. This study aimed to comprehensively summarize the clinical evidence of various traditional Chinese medicines in the treatment of DC, to explore their clinical efficacy and safety, and to explore the efficacy of traditional Chinese medicine formulations (such as decoctions, proprietary Chinese medicines, and injections) in the treatment of DC through subgroup analysis. This study provides high-quality evidence-based medical evidence for clinical diagnosis and treatment to help clinicians choose the best treatment plan.

## Author contributions

**Conceptualization:** Shuo Han.

**Data curation:** Shuo Han, Yuan Hou, Huaman Liu.

**Funding acquisition:** Huaman Liu, Quanlin Zhao.

**Methodology:** Shuo Han, Yuan Hou.

**Software:** Shuo Han, Yuan Hou.

**Supervision:** Huaman Liu, Quanlin Zhao.

**Writing – original draft:** Shuo Han.
